# Tandem Breastfeeding: A Descriptive Analysis of the Nutritional Value of Milk When Feeding a Younger and Older Child

**DOI:** 10.3390/nu13010277

**Published:** 2021-01-19

**Authors:** Elena Sinkiewicz-Darol, Urszula Bernatowicz-Łojko, Katarzyna Łubiech, Iwona Adamczyk, Magdalena Twarużek, Barbara Baranowska, Krzysztof Skowron, Diane L. Spatz

**Affiliations:** 1 Human Milk Bank, Ludwik Rydygier’ Provincial Polyclinical Hospital in Torun, 87-100 Toruń, Poland; ursber@interia.pl (U.B.-Ł.); iwona_owczarek@o2.pl (I.A.); 2 Centre of Postgraduate Medical Education, Departament of Midwifery, 01-813 Warsaw, Poland; bbaranowska@gmail.com; 3 Department of Physiology and Toxicology, Faculty of Biological Sciences, Kazimierz Wielki University, Chodkiewicza 30 St., 85-064 Bydgoszcz, Poland; k.lubiech@gmail.com (K.Ł.); twarmag@ukw.edu.pl (M.T.); 4 Department of Microbiology, Nicolaus Copernicus University in Toruń, Ludwik Rydygier Collegium Medicum, 85-067 Bydgoszcz, Poland; krzysztof.skowron@cm.umk.pl; 5 University of Pennsylvania School of Nursing, Children’s Hospital of Philadelphia, Philadelphia, PA 19104, USA; spatz@nursing.upenn.edu

**Keywords:** tandem breastfeeding, prolonged lactation, extended lactation, long-nursing mothers, child nutrition, weaning

## Abstract

Breastfeeding is a gold standard of feeding of newborns and infants. Tandem breastfeeding (TBF) is feeding two children of different ages at the same time. The knowledge about the composition of human milk in prolonged lactation is still scarce. Milk from tandem breastfeeding women and after weaning was examined. Milk samples were collected from 13 TBF mothers. A 24-h milk collection was done. Analyses of fat, protein, carbohydrate and energy content were performed using MIRIS. Sociodemographic characteristics of TBF mothers was done. Higher fat content, energy value and total protein concentration was found in TBFM milk during tandem breastfeeding, than in milk after weaning the older child. The carbohydrate content remained stable. The composition of breastmilk, in terms of macronutrients, changes after weaning, taking into account the nutritional requirements of the younger child. The milk of nursing mothers in tandem did not show diurnal variability in individual components. These findings suggest an adaptive role of human milk to nutrient requirements of newborn and older children. The results may support the promotion of long breastfeeding, including tandem breastfeeding.

## 1. Introduction

Tandem breastfeeding (TBF) is defined as the feeding of two children who are not twins. This practice is not common among mothers in either Europe or the United States [1]. Women who practice tandem breastfeeding often face criticism and social stigma. Furthermore, healthcare professionals are often unsupportive and lack knowledge about the benefits of tandem breastfeeding and the nutritional value of human milk during this practice [2,3,4]. Mothers may be told that human milk after a year is only “water” and devoid of nutritional value, or that breastfeeding an older child has no benefits. This is not research-based guidance but a current practice paradigm than can be adapted to provide evidence-based lactation education and support for families who choose to tandem breastfeed.

For women who lactate beyond a year, research demonstrates that milk has a significantly higher content of fat and energy value compared to mature milk from mothers who gave birth preterm or at full term. The protein content in milk obtained from mothers who breastfed beyond a year did not differ compared to protein levels in full term milk [5,6,7]. Human milk beyond one year postpartum also provides immunomodulatory components or antioxidant properties [8,9,10]. Although breastfeeding during pregnancy and TBF are less common, there are women who continue to breastfeed during their next pregnancy and then simultaneously breastfeed the newborn and the toddler. Research conducted among tandem breastfeeding and non-lactating mothers did not show significant differences in the composition of the colostrum produced [11]. Recommendations of scientific organizations rarely mention tandem breastfeeding, possibly only mentioning the absence of harmful effects from tandem feeding [12]. This may be due to the lack of current scientific studies on tandem feeding, or the limitations of existing studies due to the small numbers included in the study groups [13]. Existing research focuses mainly on health, psychological, or social aspects and is often qualitative research [1,14,15,16,17,18]. More research is devoted to the feeding of a child by a pregnant woman, or long feeding, where tandem feeding is not the main topic of analysis [19,20,21,22,23,24]. A review of the literature provides information that TBF does not adversely affect the health of the newborn, and tandem breast-fed children sufficiently increases body weight. TBF also does not pose a risk of maternal malnutrition, although it may cause a shortage of some nutrients [19,25,26]. Most mothers report a decrease in milk production during pregnancy [27]. Observations show that children often give up feeding during the next pregnancy because the taste of the milk changes, which is associated with hormonal changes affecting the mammary gland [28].

The primary purpose of this study was to provide descriptive statistics about tandem breastfeeding mothers (TBFM) and describe the composition of human milk in cases of women that were breastfeeding in tandem and after weaning a toddler. The scope of this research includes analysis of macronutrients including total fat, crude protein, true protein, carbohydrates, dry matter, and energy value. To the best of our knowledge, this is the first research to describe the milk composition of TBFM.

## 2. Materials and Methods

### 2.1. Study Participants

Tandem breastfeeding women were recruited via breastfeeding support groups and social media. The criterion of inclusion in the group was tandem breastfeeding, a minimum of four breastfeeding in a 24 h period. The local Ethics Committee at Warsaw Medical University accepted the information about conducting the study without reservations (AKBE/39/16). Upon entering the study, participants signed an informed consent. All study participants completed a demographic questionnaire (age, course of pregnancy, lactation period, gestational age, frequency of breastfeeding younger and older child, number of children, age of weaning, educational level, socioeconomic status, professional activity). The subjective Guttman scale was used to determine financial level: 1—low, 2—medium, 3—good.

Subjects were provided with verbal and written instructions for milk sample collection. Milk for the analysis was expressed into sterile containers and refrigerated during 24 h collection. The fresh milk was immediately transported to the laboratory of Human Milk Bank in Toruń and tested for macronutrient content.

### 2.2. Milk Sampling 

Fifty-three daily milk collection samples were obtained from 13 mothers living mostly in the Kuyavian-Pomeranian region in Poland. Sampling was performed between July 2016 and December 2017. Milk samples were collected during a 24 h period in 4 time intervals: 6:00 a.m.–12:00 a.m., 12:00 a.m.–18:00 p.m., 18:0000 p.m.–24:00 00 p.m. and 24:00 p.m.–06:00 a.m. Women expressed 5–10 mL of milk before and 5–10 mL after breastfeeding during each time interval. Milk sampling took place once a month.

### 2.3. Determination of Macronutrients

An MIRIS Human Milk Analyser (HMA™) (Miris AB, Uppsala, Sweden) was used to analyze macronutrients: total fat (g/100 mL), crude protein (g/100 mL), true protein (g/100 mL), total solids (g/100 mL) and energy content (kcal/100 mL) in milk samples. The Miris HMA is based on semi-solid mid-infrared (MIR) transmission spectroscopy. The wave ranges used in the device are specific for different groups: carbonyl (5.7 µm) for fat, amide groups (6.5 µm) for protein and hydroxyl groups (9.6 µm) for carbohydrate. A daily calibration check was performed prior to analysis using the calibration solution provided by the supplier. 

Crude protein refers to the content based on the total amount of nitrogen (N) in a sample; non-protein nitrogen compounds are also included in this value. True protein is corrected for non-protein nitrogen compounds and represents only the content of actual protein. The MIRIS HMA™ uses the factor 6.38 to convert N content to protein content. Each sample before analysis was heated at 40 °C in a thermostatic bath and then homogenized using a MIRIS Sonicator [1.5 s/mL]. Each sample was analyzed in triplicate. 

### 2.4. Statistical Analysis

The results were subjected to statistical analysis in the STATISTICA (TIBCO Software Inc., Palo Alto, California, USA. General line models and one-way ANOVA were used, treating the time of day as an independent variable and the values for the tested milk parameters as dependent variables. The significance of the differences depending on the time of day was checked using the Tukey post-hoc test at the significance level of α = 0.05. The correlation between examined milk parameters was also checked.

## 3. Results

### 3.1. Sociodemographic Characteristics of the Tandem Breastfeeding Mothers (TBFM) Group

The general characteristic of the TBF mothers group is shown in Table 1.

### 3.2. Macronutrients in TBFM Milk

The results of the analyses of nutrient content and energy value in milk samples obtained from TBF mothers are shown in Table 2.

**Chart 1:**
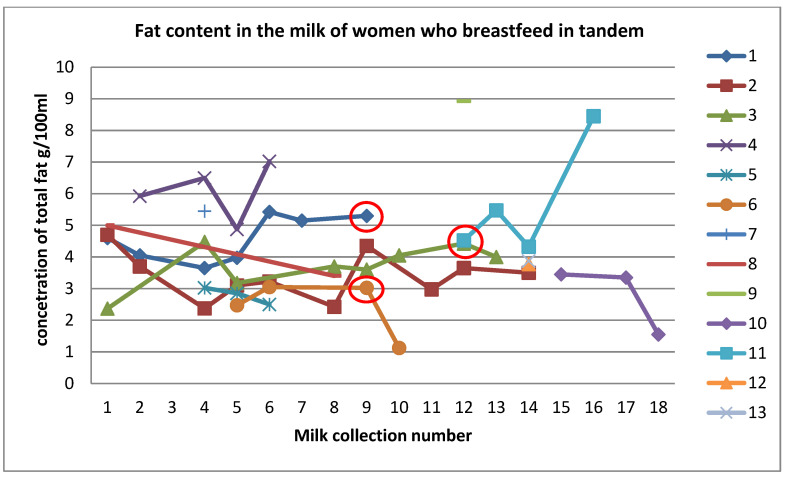
Individual changes in total fat content. The weaning time is indicated by red circle, 1–10 monthly milk collection.

**Chart 2:**
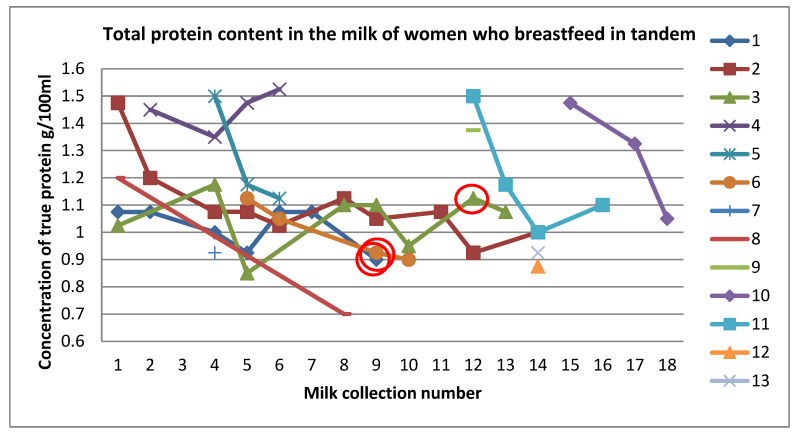
Individual changes in total protein content. The weaning time is indicated by the red circle, 1–10 monthly milk collection.

**Chart 3:**
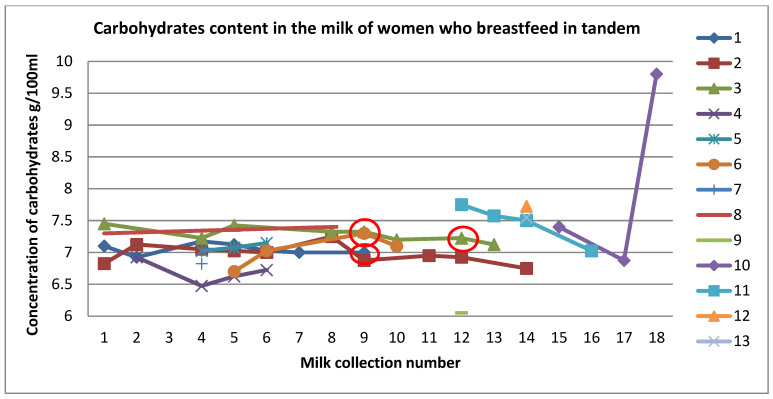
Individual changes in total carbohydrate content. The weaning time is indicated by the red circle, 1–10 monthly milk collection.

**Chart 4:**
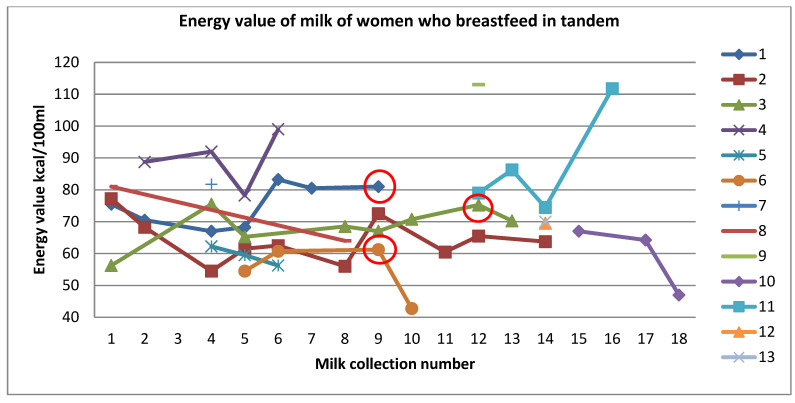
Individual changes in energy value. The weaning time is indicated by the red circle, 1–10 monthly milk collection.

The mean concentration of fat was 23.53% higher in milk samples from TBF mothers during breastfeeding of younger and older children than after weaning the toddler (3.4 ± 1.4 g/100 mL). Differences in fat concentration translates into differences in energy content, which was 14.20% higher in milk samples during TBF, compared to milk samples after weaning. The mean concentration of crude protein was 1.1 ± 0.2 (g/100 mL) in milk samples from TBF mothers. After weaning, the mean concentration of crude protein decreased by 18%. These results correlated with mean concentration of true protein, which was 0.9 ± 0.2 (g/100 mL), 0.8 ± 0.1 (g/100 mL), in groups during TBF and after weaning, respectively. There were no differences in mean carbohydrate concentration in milk samples during TBF compared to after weaning, 7.1 ± 0.4 vs. 7.1 ± 0.2 (g/100 mL) Chart 1, Chart 2, Chart 3 and Chart 4.

No statistically significant difference (*p* < 0.05) in the content of macronutrients was observed between milk samples obtained in 4 time intervals during the 24 h period. Changes in the content of individual nutrients during lactation are presented in Chart 1, Chart 2, Chart 3 and Chart 4. The time period when weaning the toddler occurred is marked by a red circle. 

### 3.3. Correlations among Macronutrients in TBFM Milk

The correlations among macronutrients of tandem breastfeeding mother’s milk are summarized in Table 3.

Statistical analysis revealed several statistically significant correlations between macronutrients. In milk samples collected from TBM, a negative correlation was observed between the carbohydrate content and the other parameters tested: total fat (−0.43), total protein (−0.18), true protein (−0.21), dry matter (−0.28) and the energy value (−0.38). All these values were statistically significant. A strong positive correlation was found between total fat and dry matter (0.99) and energy value (0.99).

### 3.4. Characteristic of Tandem Breastfeeding

The information about the decision to TBF, breastfeeding patterns and challenges are noted in Table 4.

#### Benefits and Perception of Tandem Breastfeeding

All mothers reported satisfaction and joy regarding tandem breastfeeding, despite some challenges. Most (90%) reported benefits of tandem breastfeeding such as building strong relationships and intimacy (also between children), as well as the support of the immediate family. Only one mother pointed out a lack of support from family members and only one received a positive reaction from a health care professional.

## 4. Discussion

Little research into TBF has been published in Poland and worldwide, which may be due to the small number of women who engage in the practice. The WHO has recommended breastfeeding for up to two years [29]. Thus far, there is no evidence of any adverse effects associated with breastfeeding children beyond infancy [12]. In tandem breastfeeding, feeding of the younger child must always be prioritized before feeding of the toddler, which ensures that the nutritional needs of the younger child are met. Expanding knowledge of TBF and milk composition during breastfeeding a younger and older child at the same time is critical to inform the professional health community.

There are no international data on the percentage of women who engage in tandem breastfeeding. However, Polish studies have shown that 10–18% of mothers decided to continue breastfeeding when the child turned one [30]. We showed that TBFM are well educated (94% graduate level) and are aware of the value of human milk (11.10% donate to a human milk bank). These results are compatible with previously published data from a Polish long-nursing population of mothers that reported that 85%, 91% of women breastfeeding longer than a year had a university degree [7,10]. All the children of TBFM came from a single pregnancy and were born at term (mean gestational age = 39.7 ± 0.9 weeks). The results may indicate that difficulties with giving birth prematurely are influencing the decision to continue breastfeeding [31].

In our study, the mean age of weaning was 37.7 ± 8.4 months. Sugarman (1995) showed that this is a typical weaning age in a population of long-nursing mothers [32]. Moreover, 100% of women who participated in the study reported good health of the children and that the child was introduced to solid foods according to general recommendations.

The duration of lactation influences the milk composition. It is known that during extended lactation (second or third year after post-partum), there is an increase in the content of total fat and protein. An increase in the proportion of lactoferrin, lysozyme and vitamin C was also observed. It has been shown in many studies that during the weaning process the overall concentration of protein increases. Prosser and Hartmann (1984) observed a 1.6-fold increase in protein concentration in milk from weaning women compared to non-weaning mothers [33]. They also reported a 2.8-fold increase in protein concentration in the milk from the unsuckled breast compared to a normally suckled breast. When milk volume falls below 300 mL per day, the protein content increases by 20%. In our study, we did not observe an increase in protein concentration at the time of weaning. In milk samples from TBMF, mean total protein content was 1.1 ± 0.2 (g/100 mL) and crude protein was 0.9 ± 0.2 (g/100 mL); this was 22.22% and 12.5%, respectively, higher than in milk samples after weaning. However, the protein content after weaning was at a level necessary for a younger child.

Human milk fat is characterized by high variability, both daily, during one feeding and dependent on the period of lactation [34]. Our study did not present any statistically significant differences in total fat content in milk obtained in different periods of the day (4 time intervals). We showed that mean fat content in milk samples from TBFM comprises 4.4%. After weaning, toddler fat content decreased by 19.04% to a mean concentration of 3.4 ± 1.4 (g/100 mL). This amount of fat covers the nutrient requirements of a younger child.

The most stable micronutrient in human milk is lactose. However, higher lactose levels were found in the milk of women producing higher quantities of milk [34]. Moreover, during mammary involution when milk production falls the concentration of lactose decreased 14–36% [34,35]. In our study, we did not observe any differences in lactose concentration during TBF and after toddler weaning. These observations may suggest that feeding intervals did not influence milk composition.

According to WHO recommendations, exclusive breastfeeding for the first six months of infant life is associated with improvement of nutritional status and health outcomes in children. All of women in this timely study (>6 mo) introduce complementary foods to their older or younger child. That is a greater percentage than what was previously reported in a group of breastfeeding mothers [36,37]. Most of the respondents (63%) did not plan to TBF. Bearing in mind the fact that the toddler was at the age of 34 ± 8 months and was not ready to wean, the mothers decided to continue breastfeeding (72%). All the younger infants in this study were born at term, which may have an influence on the satisfactory beginning and further course of lactation [31]. TBFM indicated the following difficulties with breastfeeding younger and older child: tiredness, frequent night waking, painful breasts, negative comments from relatives and community and mingled emotions associated with tandem breastfeeding. These observations are in consonance with previous reports devoted to breastfeeding difficulties [38]. The tiredness and frequent night waking reported by mothers were rather due to the frequency of breastfeeding of the younger child (mean 10.5 ± 3.1 per 24 h period) than the older one. In our study, only one mother heard a positive opinion about TBF from a member of healthcare personnel. The attitude of healthcare providers towards breastfeeding beyond one year often influences a mother’s decisions regarding the feeding of their children [2]. Knowledge about the composition of human milk in TBF and the health benefits of the general breastfeeding period should be the basis for healthcare professionals to support women who decide to continue breastfeeding during pregnancy and to simultaneously breast feed the younger and older children.

## 5. Conclusions

Breastfeeding for more than a year and feeding two children in tandem did not adversely affect the quality of breast milk. On the contrary, research indicates that the composition evolves as the duration of lactation increases, which is dictated by the changing needs of children. Studies in the TBM group before and after weaning indicate that the breast milk met the nutritional requirements of both the older and younger child. The composition of breastmilk, in terms of macronutrients, changes after the weaning, taking into account the nutritional requirements of the younger child. The milk of nursing mothers in tandem did not show diurnal variability in individual components. It is reasonable to promote long breastfeeding, including tandem breastfeeding.

## Figures and Tables

**Table 1:** Characteristic of tandem breastfeeding mothers group.

Maternal Characteristic	Mean ± SD
Mother’s age (years)	31.6 ± 4
Age of a younger child (months)	6.6 ± 5.1
Age of older child (months)	34 ± 8
Number of children	2 ± 0.2
Gestational age at delivery of last pregnancy (weeks)	39.7 ± 0.9
Frequency of breastfeeding an older child during 24 h period	2.7 ±1
Frequency of breastfeeding a younger child during 24 h period	10.5 ± 3.1
Age of weaning toddler (months) Total volume of milk collection during 24 h period	37.7 ± 8.4 64.5 ± 18.7
**Maternal Characteristic**	**N (%)**
The correct course of last pregnancy	yes 61% no 39%
Single pregnancy	100%
Hypertension	5.6%
Fetal hypotrophy	0%
Diabetes	5.60%
Overweight/obesity	11.10%
Anemia	11.10%
Breast milk donor	11.10%
Introduced to solid food according to general recommendation	100%
Exclusive breastfeeding (without a bottle nipple)	100%
Good children’s health	100%
**Education Level**	**N (%)**
Primary school	0%
High school complete	5.60%
University degree	94.40%
**Financial Level**	**N (%)**
Low	0%
Medium	5.60%
Good	94.40%
**Professional Activity**	**N (%)**
Yes	16.70%
No	83.30%

**Table 2:** Macronutrient and energy contents of tandem breastfeeding mothers milk and in milk samples after weaning older child.

Parameters/Groups	All Samples N = 239	Samples Obtained during TBF N = 203	Samples Obtained after Weaning Toddler N = 36
Total fat (g/100 mL)	4.1 ± 1.8 3.9	4.2 ± 1.8 3.9	3.4 ± 1.4 3.4
Crude protein (g/100 mL)	1.1 ± 0.2 1.1	1.1 ± 0.2 1.1	0.9 ± 0.1 0.95
True protein (g/100 mL)	0.9 ± 0.2 0.8	0.9 ± 0.2 0.8	0.8 ± 0.1 0.7
Carbohydrates (g/100 mL)	7.1 ± 0.3 7.1	7.1 ± 0.4 7.1	7.1 ± 0.2 7.1
Dry matter (g/100 mL)	12.5 ± 1.7 12.4	12.6 ± 1.7 12.4	11.6 ± 1.4 11.7
Energy value (g/100 mL)	71.1 ± 15.9 70	72.4 ± 16.0 70	63.4 ± 12.8 64

Results as presented as mean ±standard deviation with indication of median.

**Table 3:** The correlations among nutrients and energy value in milk samples of TBFM.

Positive Correlation						Negative Correlation
Parameters	Total Fat	Crude Protein	True Protein	Dry Matter	Energy Value	Carbohydrates
Total fat	**X**	n.s. *	0.139786 *p = 0.0096*	0.966109 *p = 0.0200*	0.987244 *p = 0.0000*	−0.431083 *p = 0.0425*
Crude protein	n.s. *	**X**	0.968509 *p = 0.0000*	0.219268 *p = 0.0001*	0.175976 *p = 0.0403*	−0.181772 *p = 0.0393*
True protein	0.139786 *p = 0.0096*	0.968509 *p = 0.0000*	**X**	0.245704 *p = 0.0030*	0.205540 *p = 0.0096*	−0.209479 *p = 0.0031*
Dry matter	0.966109 *p = 0.0200*	0.219268 *p = 0.0001*	0.245704 *p = 0.0030*	**X**	0.990120 *p = 0.0000*	−0.279513 *p = 0.0028*
Energy value	0.987244 *p = 0.0000*	0.175976 *p = 0.0403*	0.205540 *p = 0.0096*	0.990120 *p = 0.0000*	**X**	−0.375921 *p = 0.0438*

* not significant.

**Table 4:** Characteristic of tandem breastfeeding.

Why Did the Women Decide to Tandem Breastfeed?	N (%)
The toddler was not ready to wean	72%
I wanted to wait for the baby to self-wean	28%
**Did the Women Plan Tandem Breastfeeding before Last Pregnancy?**	
I did not plan TBF	63%
I considered the possibility of TBF	37%
**Breastfeeding Patterns**	
Breastfeeding on demand	46%
Breastfeeding of the older child according to a fixed ritual (e.g., at a specific time)	18%
Breastfeeding on demand after delivery younger child and then according to a fixed ritual	36%
**Difficulties with Tandem Breastfeeding**	
Tiredness, frequent night waking, painful breasts	55%
Negative comments from relatives and community	18%
Difficult, mingled emotions associated with tandem breastfeeding	9%
No problems	18%

## Data Availability

The data presented in this study are available on request from the corresponding author.
